# Looking for approved-medicines to be repositioned as anti-*Trypanosoma cruzi* agents. Identification of new chemotypes with good individual- or in combination-biological behaviours

**DOI:** 10.1590/0074-02760240183

**Published:** 2025-02-07

**Authors:** Claudia Veira, Diego Benítez, Leticia Pérez-Díaz, Guzmán Álvarez, Hugo Cerecetto, Elena Aguilera

**Affiliations:** 1 Universidad de la República, Facultad de Ciencias, Instituto de Química Biológica, Grupo de Química Orgánica Medicinal, Montevideo, Uruguay; 2 Institut Pasteur de Montevideo, Laboratory Redox Biology of Trypanosomes, Montevideo, Uruguay; 3 Universidad de la República, Facultad de Ciencias, Instituto de Química Biológica, Laboratorio de Interacciones Moleculares, Montevideo, Uruguay; 4 Universidad de la República, Centro Universitario Litoral Norte, Laboratorio de Moléculas Bioactivas, Paysandú, Uruguay; 5 Universidad de la República, Facultad de Ciencias, Centro de Investigaciones Nucleares, Área de Radiofarmacia, Montevideo, Uruguay

**Keywords:** Chagas disease, medicines-repositioning, isobologram, synergism, additivity

## Abstract

**BACKGROUND:**

The neglected illness Chagas disease is treated with limited efficacy and adverse effects by old drugs. Due to the low interest of pharmaceutical industry in targeting economically depressed-patients, repurposing is a tool that should be applied because it can introduce new anti-Chagas entities into the clinic at reduced costs.

**OBJECTIVES:**

To investigate the repurposing/combination of medicines strategies as anti-Chagas treatment.

**METHODS:**

Epimastigotes, trypomastigotes and amastigotes of *Trypanosoma cruzi* were *in vitro* exposed to 28 Uruguayan-approved medicines not previously tested, 28 FDA-approved medicines previously evaluated, and three reference agents. Parasite inhibition was assessed and for the best drugs, in pairs-isobolographic studies, looking for synergism/additivity/antagonism, were done. Macrophages were used to study selectivity. For some relevant agents, we analysed whether medicines mammals´ action mechanisms are operative in epimastigotes-*T. cruzi*.

**FINDINGS:**

From the anti-epimastigotes monotherapy-screening, we found that 18% of them showed better/comparable activities than references. Additionally, for the binary-combinations 8% were additive, 4% were synergic and the rest showed antagonism. Favourably, in macrophages-cytotoxicity four of the binary-combinations were antagonists. Naftazone and pinaverium bromide, not previously tested against *T. cruzi*, maintained their activity against trypomastigotes and amastigotes. The identified action mechanisms open the door to new strategies designing anti-*T. cruzi* drugs.

**MAIN CONCLUSIONS:**

Using approved-medicines is a good strategy for new anti-Chagas treatments.

Chagas disease (CD) is a neglected tropical disease endemic in 21 Latin American countries with tropical and sub-tropical weather, where an estimated 6 million people have chronic infection with the etiologic agent, *Trypanosoma cruzi*.^1^ The most affected populations are the ones who have low incomes and inappropriate life conditions, with constant contact with the vector that transmits these illnesses and animals acting like reservoirs. However, the disease is now a global health concern because there has been a notable increase in non-endemic countries as a result of the large-scale migration of Latin Americans to Asia, North America, Oceania, and Europe via vertical transmission from mother to child, blood transfusion or organ transplantation.^2^ ^,^ ^3^

The current treatments of CD consist of the two old nitroaromatics benznidazole and nifurtimox, which have uncertain efficacy for curing chronic infection and relevant side effects.^4^ The need for novel medicines to treat CD has not been matched by drug discovery efforts. For financial reasons, the pharmaceutical sector has made little investments in CD. Complicating the situation is the growing expense of introducing novel medicines to the market. Since the launch of benznidazole and nifurtimox in the 1960s and 1970s, no new therapeutic medicines for CD have been licensed or examined in Phase III clinical trials.^4^ ^,^ ^5^ ^,^ ^6^

Since it is extremely difficult to introduce completely new therapeutic entities through the complete drug-pipeline, various approaches to the medicines development for CD must be taken into account. On the one hand, repurposing existing medicines has been an interesting option for the identification of new drugs for CD due to the preclinical and clinical assay costs that could be avoided. In this sense, some works are landmarks in this topic, for example, the genotypic screenings performed by JA Urbina and collaborators with ergosterol biosynthesis inhibitors or by MH Gelb and collaborators with human protein prenyltransferases inhibitors.^7^ ^,^ ^8^ More recently, the phenotypic screenings of FS Buckner and collaborators involved a large number of medicines approved by the US Food and Drug Administration (FDA).^9^ Furthermore, combining of two or more bioactive agents, particularly with different mechanisms of action, is another strategy in the development of therapies for CD. There are numerous examples of polypharmacology, or multidrug therapy,^10^ where combinations of repurposed-drugs^7^ ^,^ ^11^ ^,^ ^12^ or of new research agents have been studied.^13^

Since in our country, Uruguay, many of the approved medicines have not been studied as antichagasic agents, it was thought that repositioning/combination tools could generate new therapeutic strategies or establish the basis for new anti-*T. cruzi* chemotypes. Here, we presented an *in vitro* phenotypic screening process, evaluating against epimastigotes, trypomastigotes and amastigotes of *T. cruzi*, of 28 approved medicines that were not previously tested in these formulations used in our country. Additionally, binary combinations of the best identified compounds were analysed as anti-*T. cruzi* therapies. Our findings may pave the way for further investigations on the use of repurposing/polypharmacology in the therapeutic tools for the treatment of this parasitic disease.

## MATERIALS AND METHODS

*Studied compounds* - The 56 approved medicines (trademarks, active principles and uses) and the reference agents, *i.e.*, benznidazole, ketoconazole and terbinafine, are presented in Table I and Supplementary data (Table). To extract the active principles from the solid forms of the commercially approved medicines, they were crushed and treated with ethyl acetate or dichloromethane, the excipients were filtered and the solvent was evaporated *in vacuo* using a rotary evaporator. Chromatographic and spectroscopic (NMRs) studies were carried out to study the purity of the evaporated residue. The liquid formulations were evaluated as such without extracting the active principles.

**TABLE I:** Uruguayan-approved medicines included in the current study

	Uruguayan trademark^a^	Active(s) principle(s)	Denomination
Not previously tested against *Trypanosoma cruzi*	Metorene	Naftazone	Naft
	Dicetel 100	Pinaverium bromide	Pinav
	Tizafen	Tizanidine	Tiz
	Solnef 5	Solifenacin	Solif
	Mantixa 5	Apixaban	Apix
	Sutent	Sunitinib	Sun
	Erlotinib	Erlotinib	Erlo
	Milorix	Prucalopride	Pruc
	Tamsulon	Tamsulosin	Tam
	Dublina	Ciprofibrate	Cipr
	Tadafilo	Tadalafil	Tadal
	Terina	Terazosin	Teraz
	Antivert	Somophyllin+Dihydroergocristine	Som+Dherg
	Parsidol	Profenamine	Prof
	Risperix	Risperidone	Risp
	Dumirox	Fluvoxamine maleate	Flux
	Diazepam	Diazepam	Diaz
	Delorat	Desloratadine	Deslor
	Cifloxim	Cefixime	Cefix
	Clinda	Clindamycin	Clind
	Abacavir	Abacavir sulfate	Abac
	Favipiravir	Favipiravir	Fav
	Activox	Benzydamine	Benc
	Mesacrón	Mesalazine	Mes
	Drosmina	Hidrosmin	Hid
	Flebon 500	Diosmin	Dios
	Calmasilán	*Valeriana*, *Passiflora* and *Crataegus* extract	Calm
	Placiten	*Valeriana officinalis*, *Passiflora incarnata* and *Matricaria recutita* extracts	Plac
Previously tested against *T. cruzi* in different formulations^9^	Climodín^b^	Red clover extract (*Trifolium pratense* extract)	Clim
	Plidan^c^	*Valeriana*, *Passiflora incarnata* and *Tilia* extracts+Cyclobenzaprine	Plid
	Abrilar jarabe^d^	*Hedera hélix* extract (the main component is alpha-hederin)	Abril
	Venostasin retard^e^	*Aesculus hippocastanum* Linnaeus seeds extract (the main component is Escin)	Venost
	Refren^f^	*Pelargonium sidoides* extract (main components: tanins and coumarins)	Refr
	Dipemina^g^	Diosmin+Hesperidin	Dipem
	Flodigrip^h^	Paracetamol+Caffeine+Phenylephrine hydrochloride+Loratadine	Flodi
	Desenfriol D^i^	Paracetamol+Phenylephrine hydrochloride+Chlorpheniramine maleate	Desenf
	Ataraxone^j^	Hydroxyzine hydrochloride	Atarax
	Lordex^k^	Loratadine+Dexamethasone	Lord
	Ras D^l^	Potasic losartan+Hydrochlorothiazide	Ras
	Vilzermet^m^	Metformin hydrochloride+Vildagliptin	Vilz
Used as references	Loradina	Loratadine	Lorat
	Galtoben	Gabapentin	Gaba
	Vasotenal	Simvastatin	Simv
	Furanpur	Nitrofurantoin	Nitrof
	Amxel	Amlodipine	Amlod
	Lapatinib	Lapatinib	Lapa
	Doxiciclina	Doxycycline	Dox
	Diaformina	Metformin	Met
	Kalitron	Chlorpheniramine maleate	Chlmal
	Dolex	Paracetamol	Parac
	Novemina	Dipyrone	Dipy
	Diclorazida	Hydrochlorothiazide	Hydchl
	Tendiuril	Indapamide	Indap
	Valsacor	Valsartan	Vals
	Domper	Domperidone	Domp
	Idena	Ibandronic acid	Iband
Anti-*T. cruzi* references	Benznidazol	Benznidazole	Bnz
	Etrux	Ketoconazole	Ket
	Uñex	Terbinafine	Terb

*a*: ordered within each group according to *in vitro* activity against epimastigotes; *b*: genistein, one of the extract components, was previously studied alone against epimastigotes; *c*: cyclobenzaprine was previously studied alone against epimastigotes; *d*: hederagenin (non-glycosidic alpha-hederin) was previously studied alone against epimastigotes; *e*: beta-escin was previously studied alone against epimastigotes; *f*: tannic acid and some coumarins, like 3-hydroxycoumarin, were previously studied alone against epimastigotes; *g*: hesperidin was previously studied alone against epimastigotes; *h*: each compound was previously studied alone against epimastigotes; *i*: each compound was previously studied alone against epimastigotes; *j*: it was previously studied as pamoate salt; *k*: each compound was previously studied alone against epimastigotes; *l*: each compound was previously studied alone against epimastigotes; *m*: metformin was previously studied alone against epimastigotes.

*Screening* - Entities, dissolved in dimethylsulfoxide (DMSO, Sigma) before use and then diluted in a culture milieu, were firstly tested against epimastigotes of *T. cruzi* [Tulahuen 2 strain, discrete typing unit (DTU) Tc VI] as previously described.^14^ ^,^ ^15^ Briefly, an epimastigote suspension was prepared at a concentration of 1 × 10^6^ parasites/mL, and 0.6 mL/well was inoculated into a 24-well plate incubating with a final 25 µM concentration of studied compounds. The system was incubated at 28ºC for five days and to assess cell proliferation, cell density was monitored at 610 nm at time 0 (Ap0) and five days post incubation (Ap). All measures were normalised to the control population at day 0 (Ac0) and at day 5 (Ac5). Control parasites were incubated with 0.4% DMSO, corresponding to the highest DMSO concentration of the well. The percentage of parasite inhibition was calculated as: (Ap - Ap0)/(Ac5 - Ac0) × 100. This assay was independently performed three times. For relevant compounds, the inhibitory concentration of the agent required to reduce parasite proliferation by 50% (IC_50_) was calculated by nonlinear regression analysis, fitting to a Sigmoid Boltzmann curve, using OriginLab version 8.5 for Windows. Secondly,^16^ Vero (ATCC^®^ CCL-81™) cells were infected with *T. cruzi* metacyclic trypomastigotes from 15 days old Dm28 clone (DTU Tc I) epimastigote cultures. Subsequently, the trypomastigotes harvested from this culture were used to infect further Vero cell cultures at a multiplicity of infection of 10. These trypomastigote-infected Vero cell cultures were incubated at 37ºC in humidified air and 5% CO_2_ for five to seven days. After this time, cellular trypomastigotes were collected from the supernatant by centrifugation at 3,000 × g for 10 min. The trypomastigote-containing pellets were resuspended in RPMI media supplemented with 5% foetal bovine serum (FBS) and penicillin/streptomycin at a final density of 1 × 10^8^ parasites/mL. For viability assays, 1 × 10^7^ trypomastigotes were seeded in black 96 well plates in RPMI and incubated with increasing concentrations of the entities under study for 24 h. Viability was analysed using resazurin, which is reduced to highly fluorescent resorufin. 50 μL of resazurin solution (2 mg/mL in RPMI) were added to each well and incubated for 4 h at 37ºC. Fluorescence (excitation 530 nm / emission 590 nm) was measured in a Thermo Scientific Varioskan^®^ Flash Multimode plate spectrofluorimeter instrument. Untreated parasites were used as negative controls (100% of viability). The results are presented as averages ± standard deviation (SD) of three independent biological replicates. Thirdly, for infection persistence analysis,^16^ trypomatigotes Vero cells (at a multiplicity of infection of 10) growing in RPMI were trypsinised and replated in 24-wll plates (2-3 × 10^5^ cells/well). Once adhered, the infected cells were treated with either RPMI milieu alone or milieu containing **Naft**, **Pinav** or **Terb** at different concentrations (6.25, 12.5 and 25 µM). After 24 h of incubation, cells were washed with phosphate-buffered saline (PBS), fixed with 4% paraformaldehyde, and stained with DAPI. Infected cells and amastigotes per cell were counted from randomly selected fields (400 cells per conditions). Two independent infections with trypomastigotes pre-treated with entities by two independent technicians were analysed. The percentage of uninfected Vero cells over total cells, as well as the percentage of decrease in the number of amastigotes per infected cell, was counted for each studied compound concentration tested and normalised against control untreated parasites.

*Cytotoxicity* - J774.1A macrophages (ATCC^®^ TIB67™) were grown in DMEM milieu supplemented with 10% heating-activated FBS, 1% L-glutamine, 1% streptomycin, and 100 units/mL penicillin. Cell viability was assessed using the MTT protocol. Stock solutions (at 25,000 μM) of the tested agents, 13 independent compounds, or four binary-combinations, in DMSO were prepared. Cells were grown in 96-well plates (5 × 10^4^ cells/well) for 24 h. Cultures were carried out at 37ºC in a humidified atmosphere with 5% CO_2_ and incubated with the compounds at different concentrations for 48 h. After incubation, the milieu was removed, and the cells were treated with 100 μL of 0.4 mg/mL MTT for 4 h at 37ºC. Subsequently, 100 μL of DMSO was added to the mixture. The solubilised formazan product was quantified through absorbance measurements at 570 nm. The absorbance values were transformed to the percentage of cytotoxicity compared to the negative controls.

*Binary-combination screens* - Thirteen compounds were selected for testing in binary combinations against epimastigotes, Tulahuen 2 strain, as previously described.^13^ Briefly, *T. cruzi* epimastigotes were grown at 28ºC in BHI-tryptose milieu supplemented with 5% FBS. Cells from a five-seven-day-old culture were inoculated in a fresh culture milieu to give an initial concentration of 1 × 10^6^ cells/mL. Cell growth was followed by measuring the absorbance of the culture at 600 nm every day. On day 5, the milieu was mixed with different concentrations of each compound combination dissolved in DMSO. The final concentration of DMSO in the culture milieu never exceeded 0.4%. No effect on epimastigotes growth was observed due to the presence of up to 1% DMSO in the culture milieu. Cultures containing non-treated epimastigote forms and 0.4% DMSO were included as negative controls. The different used concentrations of each compound combination were: 0.25 times IC_50,compound 1_ + 0.25 times IC_50,compound 2_; 0.5 times IC_50,compound 1_ + 0.5 times IC_50,compound 2_; 0.75 times IC_50,compound 1_ + 0.75 times IC_50,compound 2_; IC_50,compound 1_ + IC_50,compound 2_; being IC_50,compound_ previously determined. These mixtures were inoculated with 0.6 mL of a culture diluted to give a final parasite concentration of approximately 1 × 10^6^ parasites/mL. After five days the percentage of growth inhibition (PGI) was calculated for each mixture as follows: PGI (%) = {1 - [(Ap - Ap0)/(Ac - Ac0)]} × 100, where Ap = A600 nm of the culture containing the drug at day 5, Ap0 = A600 nm of the culture containing the drug just after addition of the inoculum (day 0), Ac = A600 nm of the culture in the absence of drug (control) at day 5, Ac0 = A600 nm in the absence of the drug at day 0. Then the combination values (CVs) were graphically determined and each fractional inhibitory concentration (FIC) was calculated, according to Hallander and collaborators,^17^ as the combined IC_50_ divided by the single IC_50_. The CV was defined as the concentrations of the compound combination permitting 50% inhibition (PGI = 50%). The interaction index was calculated as follows: FIC = (IC_50_ compound 1 in combination/IC_50_ compound 1 alone) + (IC_50_ compound 2 in combination/IC_50_ compound 2 alone). A FIC value less than, equal to, and more than 1 indicate synergism, additivity, and antagonism, respectively. The data were also graphically expressed as isobolograms, plotting the concentrations of each compound that combined produced a PGI = 50%. Each fractional dose was tested in triplicate and each antiproliferative experiment was done in duplicate.

*Studying whether the medicines’ mechanisms of action on T. cruzi are similar to that in mammals* - The IC_50_ values for the antiepimastigote activities were determined as it is described above modifying as follows: (i) for **Naft**, **Gaba**, **Amlod**, and **Pinav** the experiments were done in the presence of an excess of calcium, as Ca^2+^, at a final concentration of 1 mM; (ii) for **Naft** and **Gaba** the experiment was done in presence of an excess of glutamate at a final concentration of 1 mM; (iii) for **Naft** the experiment was done in presence of an excess of *N*-acetyl-L-cysteine at a final concentration of 1 mM. Additionally, nifurtimox (**Nfx**) was included in all of these assays at 25 µM. Three controls of untreated parasites and containing excess of calcium (II), glutamate or *N*-acetyl-L-cysteine (1 mM) were included. All treated and untreated wells contain no more than 0.4% of DMSO. Controls were used to calculate the relative proliferation in the treatment wells. Assays were performed in triplicate for all cases.

## RESULTS AND DISCUSSION

**Fig. 1:**
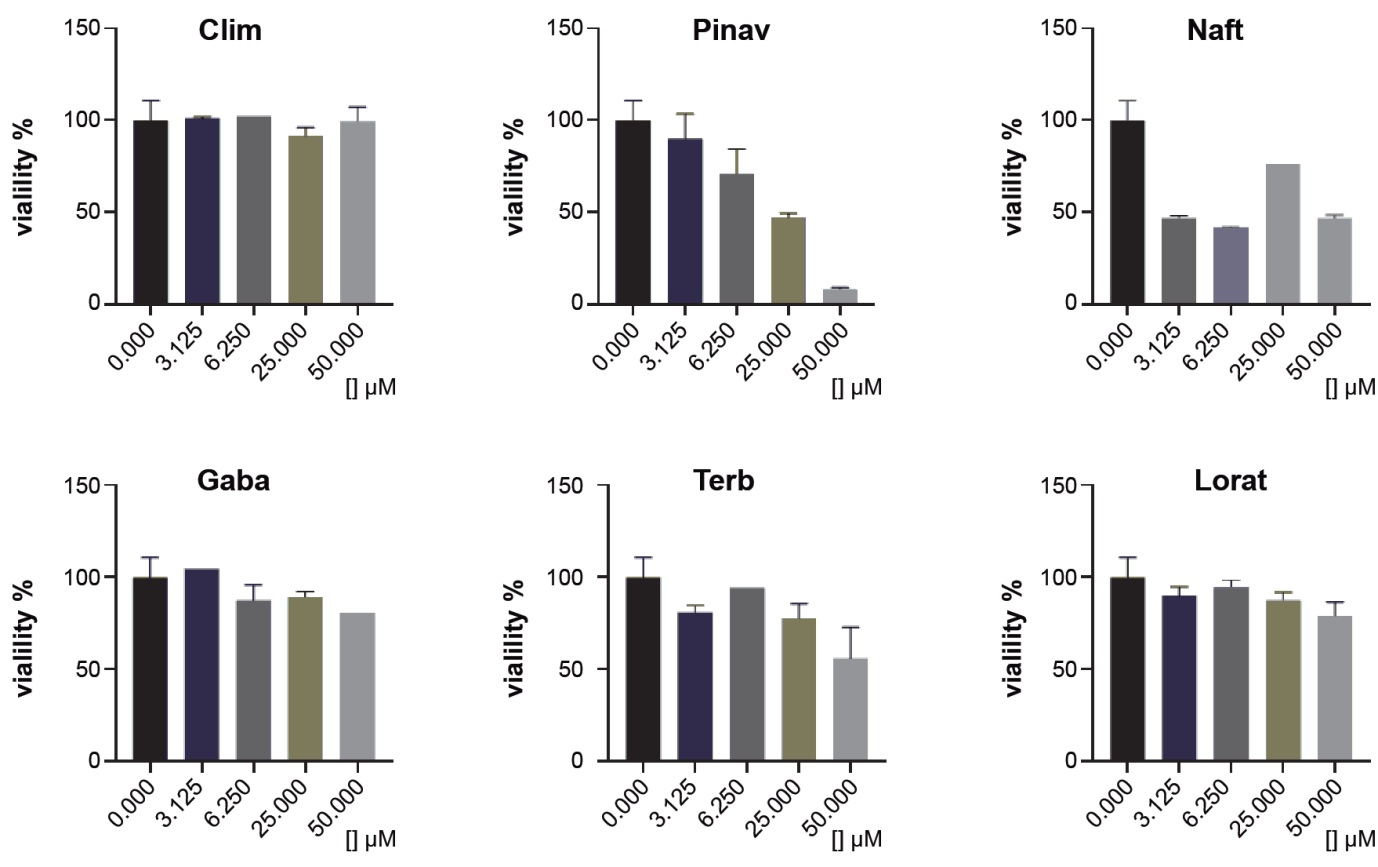
*in vitro* activity against *Trypanosoma cruzi* trypomastigotes (Dm28c) of “hit” compounds.

*Studied compounds* - We selected 28 Uruguayan-approved medicines not previously tested against *T. cruzi* to analyse as potential repurposing agents [Table I and Supplementary data (Table)]. The criteria applied for the selection of the medicines to be studied against the parasite were: (i) over-the-counter or with minimal prescription requirement in Uruguay; (ii) with wide therapeutic windows; (iii) small-molecule drugs, not biopharmaceuticals. In addition, the inclusion of drugs for orphan pathologies or pathologies with severe treatments was limited (for example some specific antitumor drugs were included but cardiotonics were not). Additionally, other 12 medicines approved in Uruguay that have already been previously studied against the parasite^9^ but in different formulations were included [Table I and Supplementary data (Table)]. In addition, another 16 medicines approved in Uruguay and previously evaluated^9^ ^,^ ^18^ were included to be used as references [Table I and Supplementary data (Table)] due to: (i) they were described as active or inactive; (ii) they cover different mechanisms of action in mammals systems [Supplementary data (Table)]; (iii) they were studied on another parasitic-strain or -stage; (iv) it wanted to compare their activity with the activity when they are administered as mixtures of some formulations commercialised in Uruguay, for example [Table I and Supplementary data (Table)] for **Lorat** and **Parac** to compare with **Flodi**, **Chlmal** and **Parac** to compare with **Desenf**, **Lorat** to compare with **Lord**, **Hydchl** to compare with **Ras** and **Met** to compare with **Vilz**. Three extra reference medicines with well anti-*T. cruzi* activities were studies, namely benznidazole (**Bnz**), ketoconazole (**Ket**) and terbinafine (**Terb**). In the cases of solid pharmaceutical forms, the corresponding active principle was extracted with organic solvent and in the case of liquid/suspension the active principle was not extracted and they were biologically evaluated in that way. The quality and purity of the extract or the composition of the liquid/suspension were analysed by chromatography or spectroscopically. As an example, in Supplementary data (Fig. 1) is shown the NMR experiment for the active medicine **Clim** [Table I and Supplementary data (Table)] which according to the manufacturer’s indications has biochanin A, formononetin, genistein and daidzein as its main components from the red clover extract.

**Fig. 2:**
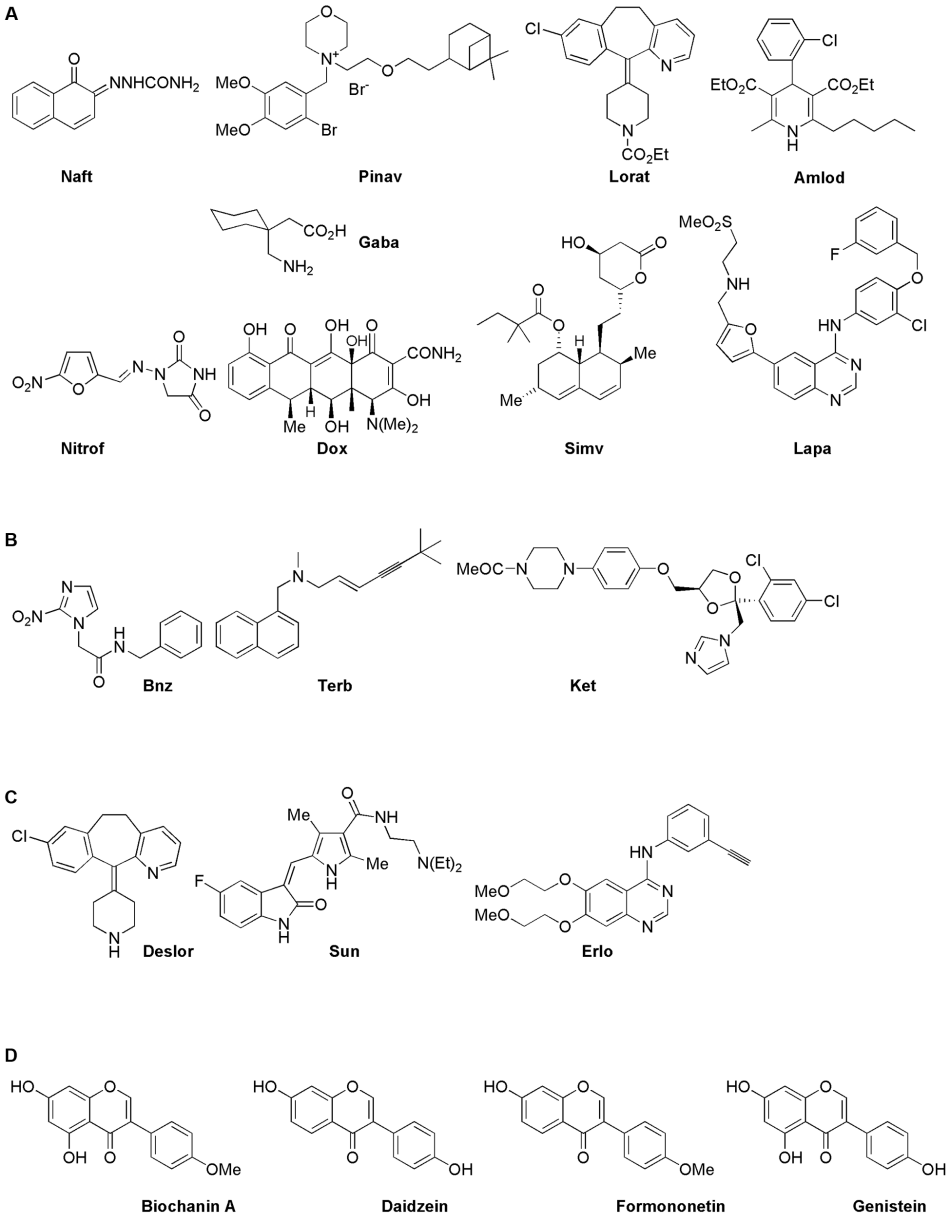
chemical structure of active principles of some medicines studied in this work. (A) Herein identified anti-*Trypanosoma cruzi* “hits”. (B) Anti-*T. cruzi* agents used as positive controls. (C) Active principles with similar mechanism of action as some entities that are “hits”, but without anti-*T. cruzi* activity detected in our study. (D) Main isoflavones detected in the herein used medicine from *Trifolium pratense* extract.

*Screening against T. cruzi and macrophages* - Initially, the 59 compounds [Table I and Supplementary data (Table)] were screened against *T. cruzi* epimastigotes, in triplicate plates, at 25 µM for extracted active principles or at 100 µg/mL for the liquid/suspension formulations. No problems of solubilities were observed during the assays. Those entities that inhibited parasitic growth by more than 65%, at these initial concentrations, were considered “hits”. In this sense, 18% of the total analysed compounds were categorised as “hits”. For them, the IC_50_s were determined in epimastigotes (Table II). The effect of these “hits” was also assayed against cellular trypomastigotes at fixed concentrations (Fig. 1) and for some of them against intracellular amastigotes [Table II, Supplementary data (Fig. 2)]. Besides, cytotoxicity against mammal systems was also analysed finding a large range of selectivity indexes (SI_epi_, Table II).

**TABLE II:** “Hits” IC_50_ against epimastigote form of *Trypanosoma cruzi*, macrophages cytotoxicities and selectivity indexes (SI), and “leads” activities against amastigote forms of *T. cruzi*

Entity^a^	Assay with epimastigotes			Assay with amastigotes		
	IC_50,epi_	IC_50,J774.1A_	SI_epi_^b^	Conc. (µM)	%ucell (%)^c,d^	%dnac (%)^e,d^
Naft	0.75 µM	101.8 µM	135.7	25	15	24
				12.5	0	0
				6.25	0	0

Bnz	4.93 µM	247.3 µM	50.2			

Lorat	0.95 µM	7.8 µM	8.21			
Amlod	13.68 µM	95.7 µM	7.00			
Nitrof	13.28 µM	61.1 µM	4.59			
Gaba	6.01 µM	19.1 µM	3.13			

Terb	21.35 µM	65.0 µM	3.05	25	-^f^	-^f^
				12.5	15	23
				6.25	4	20

Pinav	2.94 µM	8.6 µM	2.92	25	32^(***)^	21
				12.5	14^(**)^	0
				6.25	0	0

Ket	5.13 µM	13.3 µM	2.60			

Dox	19.14 µM	23.8 µM	1.24			
Simv	7.36 µM	6.5 µM	0.88			
Clim	22.70 µg/mL (80 µM^g^)	18.1 µg/mL (64 µM^g^)	0.80			
Lapa	16.60 µM	7.60 µM	0.50			

*a*: ordered according to the selectivity index on epimastigotes (SI_epi_); *b*: SI_epi_ = IC_50,J774.1A_/IC_50,epi_; *c*: %ucell = percentage of uninfected Vero cells with amastigotes respect to untreated parasites; *d*: four hundred cells were counted per experiment (n = 2) by two independent technician; *e*: %dnac = percentage of decrease in the number of amastigotes per treated Vero cell respect to control untreated infected cells; *f*: it was not evaluated due to cytotoxicity effects against Vero cell; *g*: considering the molecular mass (284.26 Da) of the main detected isoflavone, biochanin A; ANOVA Test: ^(**)^ = p < 0.01, ^(***)^ = p < 0.001.

The results against epimastigotes (Tulahuen 2 strain, DTU Tc VI) showed some relevant findings: (i) two new chemical entities displayed good-to-excellent activities, *i.e.*, **Naft** and **Pinav**. Structurally (Fig. 2) and, in principle, mechanistically, in mammalian systems, these compounds are not related. Additionally, **Naft** showed in our assays the best selectivity index, SI_epi_, being near to three-times more selective than **Bnz** (Fig. 2) while **Pinav** had a selectivity index, SI_epi_, similar to the references **Terb** and **Ket** (Fig. 2). A very interesting aspect to highlight about the active principle of **Pinav**, pinaverium bromide, is that it acts selectively blocking voltage-dependent Ca^2+^ channels in mammalian intestinal smooth muscle cells^18^ due to its permanence in the gastrointestinal tract after oral administration for its low absorption and its marked hepatobiliary excretion.^19^ Considering the parasites tropism, in some conditions, for the gastrointestinal smooth muscles, **Pinav** gastrointestinal-biodistribution could be relevant for digestive form of the CD; (ii) In our hands, the second repositioned-medicine with excellent activity against epimastigotes and with correct SI was **Lorat** (Fig. 2). Previous studies,^11^ on other strains/clone (Y, Dm28c and CL Brener), did not find such excellent **Lorat** activity on epimastigotes (compared to **Bnz**) but did find similar SI_epi_ values. An interesting aspect is that other pharmaceutical formulations that include the active principle of **Lorat**, *i.e.*, **Flodi** and **Lord**, were not active, the same occurred as the loratadine desethoxycarbonyl-derivative, **Deslor** (Fig. 2), which did not show anti-epimastigote activity; (iii) Other entities that gave good results in activity and selectivity were **Amlod** and **Gaba** (Fig. 2), which were previously evaluated^9^ ^,^ ^20^ and chosen in this work by mammals-mechanistic analogy with **Pinav**. **Amlod** displayed the highest selectivity however **Gaba**, like **Pinav**, had similar selectivity that **Terb** and **Ket**; (iv) Other identified anti-epimastigotes “hits” were the previously described **Nitrof**,^9^ ^,^ ^21^ ^,^ ^22^ **Dox**,^9^ ^,^ ^12^ **Simv** ^8^ ^,^ ^9^ and **Lapa** ^23^ (Fig. 2) however they have different selectivity degrees being **Nitrof** and **Dox** more cytotoxic for epimastigotes and **Simv** and **Lapa** for the mammal cells model tested. Other entities with mammals-mechanistic similarities with **Lapa**, *i.e.*, **Sun** and **Erlo** (Fig. 2), did not display anti-*T. cruzi* activities in the assayed conditions; (v) The red clover (*Trifolium pratense*) extract, *i.e.*, **Clim**, displayed relevant anti-*T. cruzi* activity but lower selectivity. According to the manufacturer, this isoflavones’ extract has biochanin A, daidzein, formononetin, and genistein (Fig. 2) as its main components. Our NMR experiments [Supplementary data (Fig. 1)] of the herein-used product indicated the following approximate main composition: 65% of biochanin A, 15% of daidzein, 10% of formononetin and 10% of genistein. Previously, genistein was described as anti-*T. cruzi* agent^9^ and other kinds of clover (*Trifolium clypeatum* Linnaeus) extract as anti-*Leishmania* agent^24^ were evaluated with similar results herein observed.

The rest of the medicines evaluated (Table I), with different therapeutic indications [Supplementary data (Table), for example, vasoprotectors, analgesics, anti-inflammatories, antivirals, antibacterials, vasodilators, among others], did not surpass the first cut-off criterion against epimastigotes.

From these results and the isobolographic analysis (see next section, Table III), a group of “leads” emerged, *i.e.*, **Naft**, **Lorat**, **Gaba**, **Terb**, **Pinav** and **Clim**, that were evaluated against the trypomastigote form of the parasite. In this study **Naft** and **Pinav** emerged again as entities of interest, the former being an excellent cytotoxic agent for this form of the parasite (Fig. 1). The rest of the studied entities, except **Terb**, did not display relevant anti-trypomastigote activities at the studied doses.

**TABLE III:** Results of the biological studies of the binary-combinations that displayed additivity or synergistic effects against *Trypanosoma cruzi* epimastigotes

Binary-combination	Result against *T. cruzi*	FIC_epi_	Result against J774.1A	FIC_J774.1A_	Compound-concentrations at the corresponding IC_50,J774.1A_
Bnz+Terb	Additive	1.10	Antagonistic	1.46	[Bnz] = [Terb] = 75.10 µM
Bnz+Ket		0.99	n.s.^a^	n.s.	n.s.
Naft+Ket		1.09	Additive	1.04	[Naft] = [Ket] = 12.24 µM
Naft+Gaba		1.04	Antagonistic	6.36	[Naft] = [Gaba] = 101.65 µM
Naft+Clim		1.07	Synergistic	0.45	[Naft] = 6.89 µM, [Clim] = 24.2 µM
Terb+Simv		1.07	Synergistic	0.12	[Terb] = [Simv] = 0.65 µM

Lorat+Terb	Synergistic	0.73	Antagonistic	2.83	[Lorat] = [Terb] = 19.55 µM
Nitrof+Ket		0.69	Synergistic	0.45	[Nitrof] = [Ket] = 4.9 µM
Clim+Lapa		0.90	Antagonistic	1.68	[Clim] = 31.60 µM, [Lapa] = 8.99 µM

*a*: not studied.

Finally, to assess the persistence of the parasite in an established infection, pre-infected monolayers (without prior exposure to studied entities) were treated with the corresponding “leads”, *i.e.*, **Naft** and **Pinav**, together **Terb** as positive control, at different concentrations [Table II, Supplementary data (Fig. 2)]. The number of amastigotes per cell as well as the number of infected cells was then counted. A significant reduction on the number of infected cell was observed for **Pinav** treated cells at 25 and 12.5 µM and a tendency on the number infected cell for **Naft** treated cells at 25 µM. Additionally, tendencies to a decrease in intracellular parasites was observed after 24 h of incubation using concentrations of 25 µM for **Pinav** and **Naft**. This suggests that these “leads” can be able to inhibit the replication of infected cells as well as the proliferation of intracellular parasites in a dose-dependent manner.

*Binary-combination studies* - The thirteen compounds of Table II were selected for testing in binary-combinations against Tulahuen 2 epimastigotes using isobolographic studies. Therefore, it was found that six of the binary combinations were additive (~ 8% of the total studied binary-combinations) and three (~ 4% of the total studied binary-combinations) of them were synergistic (Table III). The results showed interesting binary-combinations that could guarantee promising therapeutic uses due to different mechanisms of action, in mammal systems, of their entities, *i.e.*, blockage of nucleic acid synthesis (**Bnz**), effects on the sterol-membrane biosynthesis (**Terb**, **Ket**, **Simv**), stabilisation of the lysosomal membrane (**Naft**), blockade of calcium ion channels (**Gaba**), action on oestrogen receptors (**Clim**), blockade of histamine H1 receptors (**Lorat**), inhibition of protein synthesis (**Nitrof**) or blockage of tyrosine kinases receptors (**Lapa**).

Our study also provides interesting information about those binary combinations that will be antagonistic in the therapeutic action and therefore it would not be advisable to continue with subsequent *in vivo* studies. Some findings previously described could be explained by our results of antagonism. For example, the *in vitro* antagonism observed herein between **Bnz** and **Dox** would explain the lack of additive *in vivo* activity previously described with this binary-combination.^12^

Additionally, our findings could explain some previous studies. For example, our *in vitro* results of additivity between **Bnz** and **Ket** are partially in agreement with the *in vivo* results observed by Z. Brener and collaborators with this combination.^24^ These authors found that some treatments with the combinations of these entities produced complete cure of animals infected with CL Brener clone (DTU Tc VI), partial cure of animals infected with Y strain (DTU Tc II) and absence of cure of animals infected with Colombiana strain (DTU Tc I). Another interesting aspect that our findings could highlight is the inconsistency of previous result in the binary combination **Ket** and **Terb** and our result. Previously J.A. Urbina and collaborators^7^ ^,^ ^25^ found *in vitro* and *in vivo* synergism of this binary-combination against different *T. cruzi* strains, one isolated from a paediatric patient and another of DTU Tc II (Y strain), while in our work a different strain was used (Tulahuen 2 strain, DTU Tc VI). All these divergences could be indicating the relevance of the genotype of the strain under study in the biological response.

The cytotoxicity against mammal system, J774.1A macrophages, was also analysed for the binary-combinations with anti-*T. cruzi* additive or synergistic effects (Table III). Since the binary-combination of **Benz** and **Ket** has already been studied *in vivo*,^24^ it was not included in these *in vitro* cytotoxicity-studies. In the cytotoxicity on macrophages the optimal result to continue studying the binary-combination is for it to show antagonism or eventually additivity while if the binary-mixture produced synergism against macrophages, it implies that the mammalian-cytotoxic effects are enhanced. Regarding the results, they were highly satisfactory for four of the binary-combinations that were antagonistic. The binary-combination **Naft**+**Gaba**, which was additive against *T. cruzi*, showed a clear antagonistic process towards mammalian cells (FIC = 6.36, Table III), a fact that is highly relevant for thinking in subsequent *in vivo* studies of this binary-combination. Similarly, but with a lower FIC, was the additive binary-combination **Bnz**+**Terb** (FIC = 1.46, Table III). The anti-*T. cruzi*-synergistic binary-combinations **Lorat**+**Terb** and **Clim**+**Lapa** showed also antagonisms against J774-1A mammalian system, with FIC of 2.83 and 1.68, respectively (Table III), which could indicate the absence of extra toxic effects when using this binary-combination *in vivo*. On the other hand, the binary-combination **Naft+Ket** was additive against macrophages, adding the cytotoxicity of each independent entity. The rest of the studied binary-combinations, **Naft+Clim**, **Terb+Simv,** and **Nitrof+Ket**, were synergistic therefore cytotoxic effects in mammals are enhanced compared to the cytotoxic effects of each independent entity.

*Study of some medicines’ mechanisms of action on T. cruzi* - Here we have focused on those medicines’ mechanisms of action that could be partially novel or novel for the development of future anti-*T. cruzi* agents, namely: (i) inhibition of Ca^2+^ parasitic-ion channels; (ii) change in the glutamate parasitic-concentration; (iii) parasitic-lysosomal membrane’ stabilisation. Regarding the inhibition of Ca^2+^ channels, **Gaba**,^26^ **Amlod** ^27^ and **Pinav** ^28^ have been described as having this type of mechanism of action on mammalian cells by inhibition of this kind of cationic channel. On the other hand, for **Naft**, and a **Naft** glucuronide-metabolite, or **Gaba** it was described the ability of these compounds to decrease the release of glutamate for synaptosomes isolated from the cerebellum of mouse brain^29^ or in the presynaptic terminals in the spinal cord,^26^ respectively. Additionally, **Naft** has been described to act as a lysosomal membrane’ stabiliser and this mechanism of action is related to different proposed facts: (i) the control of oxidation-reduction reactions by **Naft** of lysosomes membranes;^30^ (ii) the inhibition of cellular Ca^2+^ uptake, via **Naft** glucuronide-metabolite,^29^ having Ca^2+^ a critical play in the glutamate induced-lysosomal membrane permeability.^31^

To validate these targets for the different studied medicines, we used indirect strategies evaluating the mechanisms of **Naft**, **Gaba**, **Amlod,** and **Pinav** on *T. cruzi* through the measurement of IC_50_ in the presence of excess or absence (control) of: (i) calcium cation for **Naft**, **Gaba**, **Amlod** and **Pinav**; (ii) glutamate for **Naft** and **Gaba**; (iii) and *N-*acetyl-L-cysteine for **Naft**.

If the calcium channels are involved in the anti-*T. cruzi* activity of **Naft**, **Gaba**, **Amlod,** and **Pinav**, an increase in the IC_50_ of those entities in the presence of Ca^2+^ excess is expected. The results showed an increase of IC_50_ values of at least 3.7 times when an excess of Ca^2+^ was added to the treated epimastigote *T. cruzi* with all the studied entities (Table IV). As expected, the percentage of growth inhibition of **Nfx** was not affected by the addition of excess of Ca^2+^ to the treated parasites (Table IV). For **Gaba**, **Amlod** and **Pinav** a complete loss of activity at the assayed doses was evidenced showing that the blockade of calcium ion channels could be also operative in *T. cruzi* as the mechanism of action. On the other hand, **Naft** continued to be active although the IC_50_ increased almost four times in the presence of Ca^2+^, consequently, the activity of this entity seems to be attributed in part to this mechanism but another mechanism of action must be operative.

**TABLE IV:** *In vitro* anti-*Trypanosoma cruzi* activities, expressed as IC_50_ (µM) and as IC ratio (r_IC_) between the IC_50_ with and without the excess of added reagent, of some entities herein studied in a modified biological milieu. **Nfx** was studied at a fixed dose (25 µM) expressing the activity as a percentage of growth inhibition at 25 µM

Entity	excess of Ca^2+^		excess of glutamate		excess of *N*-acetyl-L-cysteine (NAC)	
Naft	IC_50,epi_ = 2.8	r_IC_ = 3.7	IC_50,epi_ = 13.5	r_IC_ = 18.0	IC_50,epi_ = 1.9	r_IC_ = 2.5
Gaba	IC_50,epi_ > 25.0	r_IC_ > 4.2	IC_50,epi_ > 25.0	r_IC_ > 4.2		
Amlod	IC_50,epi_ ~ 25.0	r_IC_ ~ 1.8				
Pinav	IC_50,epi_ > 25.0	r_IC_ > 8.5				
Percentage of growth inhibition at 25 µM (%)						
Nfx	without Ca^2+^	with Ca^2+^	without Glu	with Glu	without NAC	with NAC
	70	61	70	55	74	20

On the other hand, due to the involvement of glutamate in different processes promoted by **Naft** and **Gaba**, affecting the mammals-lysosomal membrane in the case of **Naft** or decreasing its release from mammal cells in the cases of **Naft** and **Gaba**, parasites’ inhibition studies with excess of glutamate were carried out. In these cases, the IC_50_s increased for both entities, which notably did not occur with the percentage of growth inhibition promoted by **Nfx** (Table IV). These results could indicate that the change in the glutamate parasitic-concentration, specifically depletion, is related to anti-parasite activities of both entities. For **Gaba** again the complete loss of activity, at the assayed doses, showed that this mechanism of action could be clearly operative in *T. cruzi*. Particularly, in the case of **Naft**, in the presence of glutamate excess, it continued to be active but the IC_50_ increased 18 times in the presence of glutamate, consequently, the activity of **Naft** seems to be attributed primarily to the glutamate homeostasis.

Finally, if **Naft** acts via the reduction-oxidation process, as it is proposed for mammals by modifying the lysosomal membrane permeability, an increase in the IC_50_ of **Naft**-treated-parasite in the presence of an excess of the recognised antioxidant *N*-acetyl-L-cysteine^31^ is expected. Clearly, the IC_50_ value increased just 2.5-fold when this antioxidant was added (Table IV), consequently the reduction-oxidation **Naft** participation seems to be a secondary mechanism of action. As expected, the percentage of growth inhibition of **Nfx** was clearly affected by the addition of excess of *N*-acetyl-L-cysteine to the treated parasites (Table IV) confirming the well-proposed mechanism of action of this drug.^32^ Then, we concluded that the antiepimastigote response of **Naft** could be also in part derived from a reduction-oxidation process, probably like in mammal cells affecting the permeability of the membrane of lysosome-like compartments (or the epimastigote reservosomes).^33^

For the best herein repositioned medicine, *i.e.*, **Naft**, the anti-*T. cruzi* role is probably mediated through the cross-talk mechanism of actions between Ca^2+^ inhibition uptake, glutamate homeostasis modification and redox-processes maybe being the *T. cruzi-*lysosome-like membrane permeability the final target. This mode of action must be studied deeply in the future.
