# Supplementary material for: Looking for approved-medicines to be repositioned as anti-Trypanosoma cruzi agents. Identification of new chemotypes with good individual- or in combination-biological behaviours
Source: Mem Inst Oswaldo Cruz. 2025 Feb 7;120:e240183. doi: 10.1590/0074-02760240183 (PMC11809512; doi:10.1590/0074-02760240183)
Supplement: Supplementary file 1 [file 1678-8060-mioc-120-e240183-s.pdf]

TABLE

Denomination	Active(s) principle(s)	Use in therapy		Mechanism or mode of action in mammals
Naft	Naftazone	To treat of varicose veins, hemorrhoids and venous diseases<br>Inhibits the breakdown of noradrenaline by catechol-*O*-methyltransferase<br>Influences on the permeability and fragility of microvessels, increases parietal tone and increases erythrocyte deformability and reduces the viscosity of the blood<br>Hesperidin inhibits human Aurora kinase HsAurB inhibitor		Stabilisation of capillary walls, anti-inflammatory actions, improvement of blood rheology, protection of endothelial cells from oxidative stress and modulation of enzymatic activities
Dios	Diosmin			
Hid	Hidrosmin			
Dipem	Diosmin+Hesperidin			
Pinav	Pinaverium bromide	To restore the normal contraction process of the bowel (antispasmodics by Ca^{2+} channel blocker)		Modifiers of Ca^{2+} homeostasis
Gaba	Gabapentin	To treat partial seizures and neuropathic pain (anticonvulsant)		
Amlod	Amlodipine	To treat coronary artery disease, variant angina and high blood pressure		
Tiz	Tizanidine	To treat muscle spasticity due to spinal cord injury, multiple sclerosis, and spastic cerebral palsy		Agonist of α2-adrenergic receptor
Solif	Solifenacin	To treat overactive bladder and neurogenic detrusor overactivity		Competitive cholinergic receptor antagonist (selective for the M3 receptor subtype)
Apix	Apixaban	To treat and prevent blood clots and to prevent stroke in people with nonvalvular atrial fibrillation (anticoagulant)		Reversible direct inhibitor of free and clot-bound factor Xa
Sun	Sunitinib	To treat cancer		Targets receptor tyrosine kinase
Erlo	Erlotinib			
Lapa	Lapatinib			
Pruc	Prucalopride	To treat the impaired motility associated with chronic constipation		Selective $5\text{-}HT_4$ receptor agonist
Tam	Tamsulosin	To treat symptomatic benign prostatic hyperplasia, chronic prostatitis and to help with the passage of kidney stones		Selective α1 receptor antagonist
Cipr	Ciprofibrate	To treat hyperlipidemias<br>Competitive and reversible inhibitor of HMG CoA reductase		Binds to PPARα, increasing fatty acid oxidation and reducing triglycerides levels
Simv	Simvastatin			
Prof	Profenamine	Action on the CNS	To treat Parkinson disease	Anticholinergic, antihistamine, and antiadrenergic actions
Risp	Risperidone		To treat schizophrenia and bipolar disorder	Serotonin and norepinephrine reuptake inhibition
Flux	Fluvoxamine maleate		To treat, among others, major depressive disorder and obsessive–compulsive disorder	Selective serotonin reuptake inhibitor
Diaz	Diazepam		To treat, among others, anxiety, seizures, muscle spasms and insomnia	Binds to the benzodiazepine site on the GABAA receptor
Tadal	Tadalafil	Vasodilators	To treat erectile dysfunction, benign prostatic hyperplasia, and pulmonary arterial hypertension	Releases NO stimulating the synthesis of cyclic guanosine monophosphate in smooth muscle cells
Teraz	Terazosin		To treat symptoms of an enlarged prostate and high blood pressure	Blocks alpha-1-selective adrenergic receptors
Som+Dherg	Somophyllin+Dihydroergocristine		To vasodilate cerebral vessels	Agonistic activity on dopaminergic and antagonistic activity on adrenergic receptors
Ras	Potasic losartan+Hydrochlorothiazide		To treat hypertension	Losartan is an angiotensin receptor blocker
Hydchl	Hydrochlorothiazide		To treat hypertension and swelling due to fluid build-up	Inhibits the NaCl cotransporter located on the apical membrane of the distal convoluted tubules in the kidney
Indap	Indapamide		To treat hypertension and decompensated heart failure	Diuretic effect at the level of the kidney distal tubule and a direct vascular effect
Vals	Valsartan		To treat hypertension, heart failure and diabetic kidney disease	Angiotensin II receptor blockers

CC BY

Denomination	Active(s) principle(s)	Use in therapy		Mechanism or mode of action in mammals
Deslor	Desloratadine	Antiallergic	To treat allergies	H_1 inverse agonist
Lord	Loratadine+Dexamethasone		Dexamethasone: to treat, among others, severe allergies	Dexamethasone alleviates allergic inflammation through an MKP-1-dependent mechanism
Lorat	Loratadine		To treat allergies	H_1 receptor inverse agonist
Atarax	Hydroxyzine hydrochloride		To treat, among others, itchiness	
Cefix	Cefixime	Antibiotic	To treat different bacterial infections	Inhibition of cell wall synthesis
Clind	Clindamycin			Inhibits bacterial protein synthesis
Dox	Doxycycline		To treat bacterial and certain parasites infections	
Nitrof	Nitrofurantoin		To treat urinary tract infections	Affects, among others, ribosomal proteins, DNA, respiration, pyruvate metabolism
Abac	Abacavir sulfate	Antiviral	To treat HIV/AIDS	Reverse-transcriptase inhibitor
Fav	Favipiravir		To treat, among others, influenza and SARS-CoV-2	Inhibits viral RNA-dependent RNA polymerase
Benc	Benzydamine	Analgesic/anti-inflammatory/antipyretic/anti-allergic<br>Decreases synthesis of prostaglandin and leukotriene<br>Caffeine: increases intracellular concentrations of cyclic adenosine monophosphate<br>Phenylephrine: stimulates postsynaptic alpha-receptor<br>Selective inhibitor of histamine H1 receptors<br>Activates the descending serotonergic pathways<br>Inhibits prostaglandin E2-induced hyperalgesia		Inhibits the synthesis of pro inflammatory cytokines (i.e., TNF-α, IL-1β)
Mes	Mesalazine			
Flodi	Paracetamol+Caffeine+Phenylephrine hydrochloride+Loratadine			
Desenf	Paracetamol+Phenylephrine hydrochloride+Chlorpheniramine maleate			
Chlmal	Chlorpheniramine maleate			
Parac	Paracetamol			
Dipy	Dipyrone			
Calm	*Valeriana*, *Passiflora* and *Crataegus* extract	Natural products extracts<br>Anxiolytic, antispasmodic and sleep promoter<br>Action on climacteric symptoms acting on on type b estrogen receptors<br>Anxiolytic<br>Effect on bronchial secretions<br>Effect on the venous system<br>Antibacterial, antiviral and immunomodulatory		Tranquiliser and sleep normaliser
Plac	*Valeriana officinalis*, *Passiflora incarnata* and *Matricaria recutita* extracts			
Clim	Red clover extract (*Trifolium pratense* extract)			
Plid	*Valeriana*, *Passiflora incarnata* and *Tilia* extracts+Cyclobenzaprine			
Abril	*Hedera hélix* extract (the main component is alpha-hederin)			
Venost	*Aesculus hippocastanum L.* seeds extract (the main component is Escin)			
Refr	*Pelargonium sidoides* extract (main components: tanins and coumarins)			
Vilz	Metformin hydrochloride+Vildagliptin	To treat diabetes<br>Inhibits, among others, mitochondrial respiratory chain, glucagon-induced elevation of cyclic adenosine monophosphate, mitochondrial glycerol-3-phosphate dehydrogenase		Inhibits dipeptidyl peptidase-4
Met	Metformin			
Domp	Domperidone	To treat nausea and vomiting and certain gastrointestinal problems		Dopamine antagonist

Denomination	Active(s) principle(s)	Use in therapy	Mechanism or mode of action in mammals
Iband	Ibandronic acid	To prevent and treat osteoporosis and metastasis-associated skeletal fractures in some cancers	Inhibits farnesyl pyrophosphate synthase and binds to and adsorbs onto the surface of hydroxyapatite crystals in the bone matrix
Bnz	Benznidazole	Anti-*T. cruzi* references	-
Ket	Ketoconazole	-	
Terb	Terbinafine	-	

Codes

	Not previously tested against *Trypanosoma cruzi*
	Some components, but not all or in the combination, or other chemical form (*i.e.*, salt) of the active principle of the Uruguayan medicines formulations were previously evaluated against *T. cruzi*
	Used as references due to: (i) they were described as active or inactive; (ii) they cover different mechanisms of action in mammals systems; iii) they were studied on another parasitic-strain or -stage
	Used as references in order to compare their activity with the activity when they are administered as mixtures of some medicines formulations commercialised in Uruguay
	Anti-*T. cruzi* reference agents

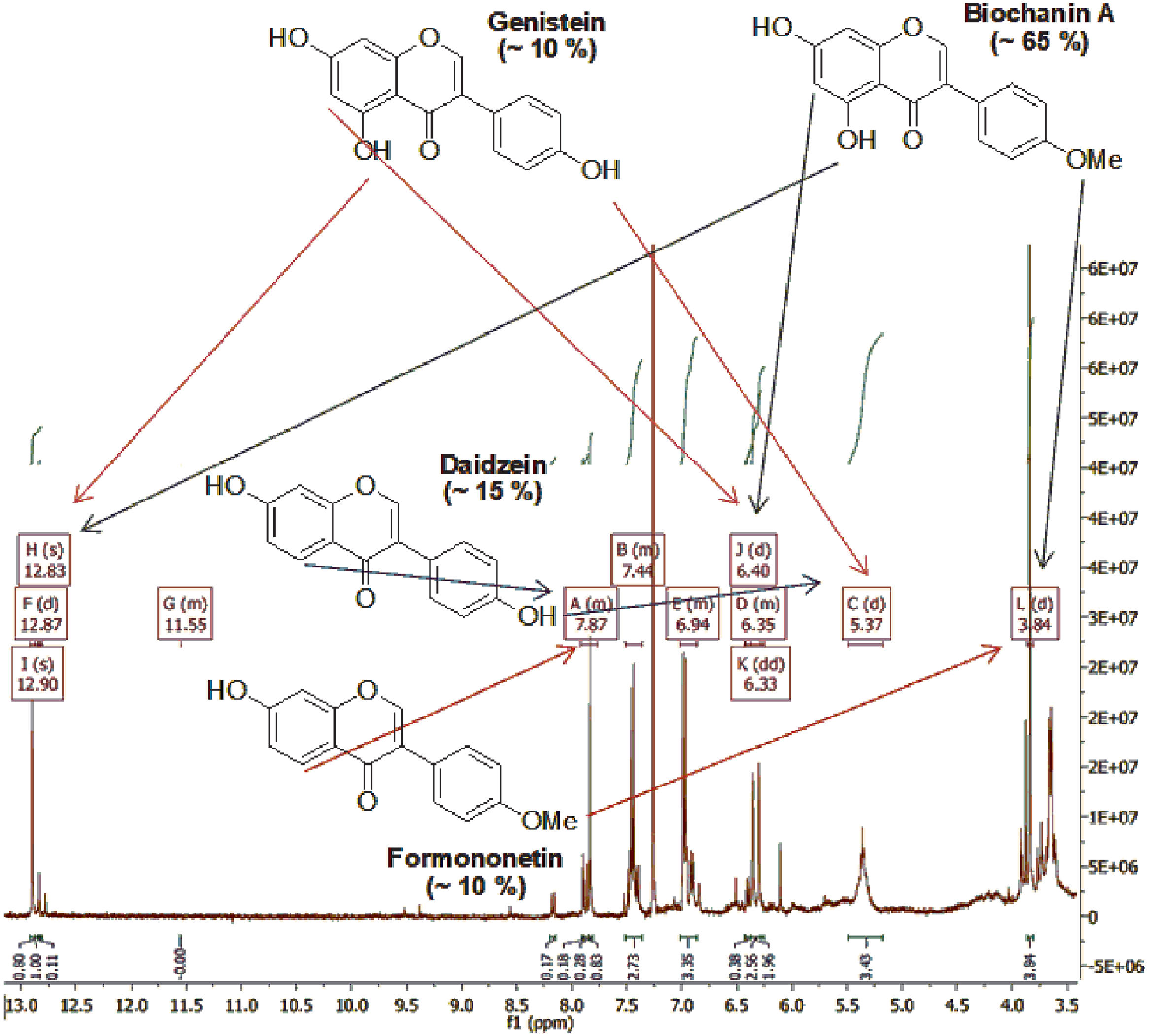

Fig. 1: 1H NMR of the active medicine Clim (CDCl3, room temperature) which according to the manufacturer's indications has biochanin A, formononetin, genistein and daidzein as its main components from the red clover extract. Selected signals to calculate the proportion of each compound are indicated.

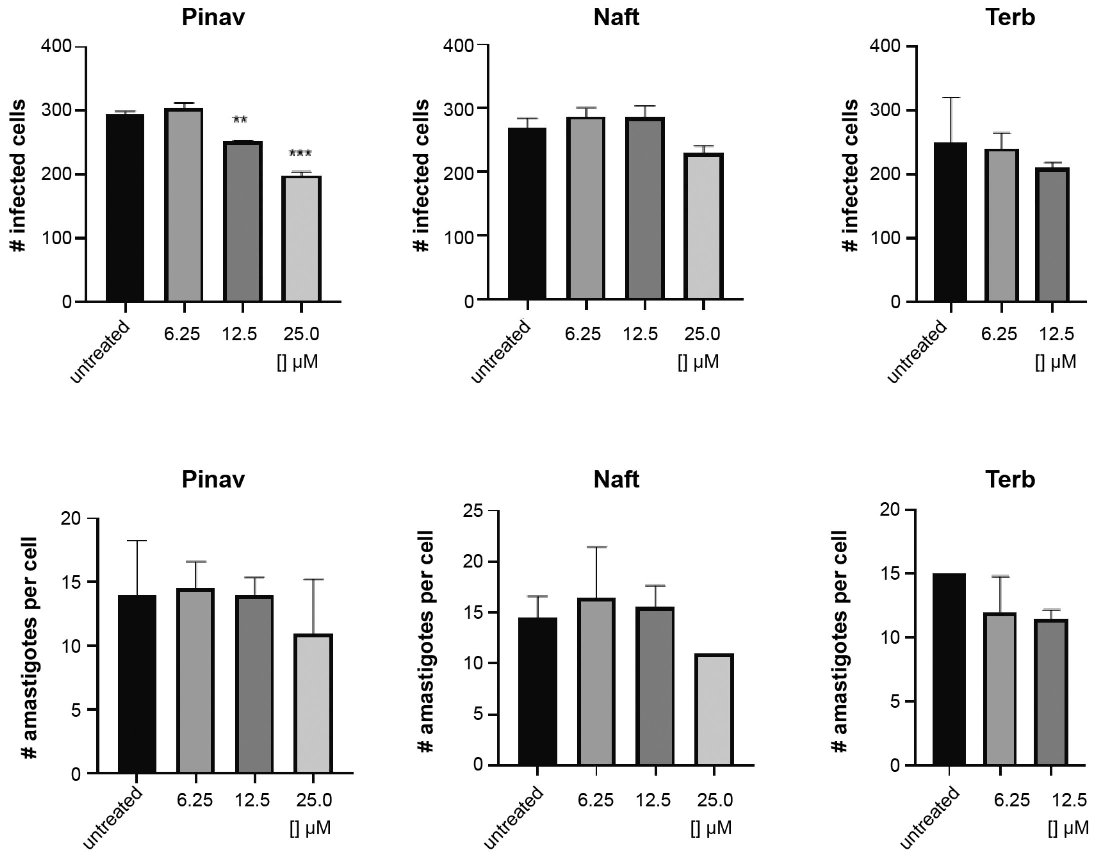

Fig. 2: effect of Pinav, Naft and Terb on infection persistence. Up: Number of infected cells after 24 hours of treatment with the indicated concentrations of the indicated entities. Down: Number of amastigotes per cell on already infected cells incubated with different concentrations of the indicate entities after 24 hours of incubation. Two independent experiment were performed. In each experiment, at least 400 cells were counted per experiment. ANOVA Test: (**) = $p < 0.01$, (***) = $p < 0.001$.
